# Manipulation in Stricture

**Published:** 1874-11

**Authors:** 


Manipulation in Treatment of Stricture.—M. Bardinet, of Limoges, in the treatment of stricture, strongly recommends what he calls the "*massage*" of the urethra. When the bougie has become engaged within the stricture, and would in ordinary practice be so left immovable for a longer or shorter time, he deals with it otherwise. Seizing the penis gently with the left hand, he takes hold of the bougie in the stricture, and impresses upon it repeated to and fro movements, varying from ten to thirty in number, and gradually increasing in amplitude. The bougie, so tightly held at first, becomes free, and moves with astonishing facility. One a little larger is then introduced, and the same procedure repeated; and a third may follow this. In fact, in a short time a considerable amount of dilatation has been accomplished; and the treatment of the case is much abridged by this procedure.—*Union Med., August* 20, 1874.

